# The Ulaanbaatar agreement: Revising diabetes terminology in Asia to combat stigma

**DOI:** 10.1111/jdi.14330

**Published:** 2024-10-03

**Authors:** Yutaka Seino, Daisuke Yabe, Kazuhiro Tsumura, Chien‐Ning Huang, So Hun Kim, Weiping Jia, Altaisaikhan Khasag, Takashi Kadowaki

**Affiliations:** ^1^ Kansai Electric Power Hospital Osaka Japan; ^2^ Kansai Electric Power Medical Research Institute Kyoto Japan; ^3^ Kyoto University Graduate School of Medicine Kyoto Japan; ^4^ Center for Metabolism and Clinical Nutrition Kawasaki Municipal Hospital Kawasaki Japan; ^5^ Department of Internal Medicine Chung Shan Medical University Hospital Taichun Taiwan; ^6^ Division of Endocrinology & Metabolism Inha University College of Medicine Incheon Korea; ^7^ Shanghai Diabetes Institute, Shanghai Key Laboratory of Diabetes Mellitus, Shanghai Key Clinical Center for Metabolic Diseases Shanghai Jiao Tong University Affiliated Sixth People's Hospital Shanghai China; ^8^ Department of Internal Medicine, School of Medicine Mongolian National University of Medical Sciences Ulaanbaatar Mongolia; ^9^ Toranomon Hospital Minato‐ku, Tokyo Japan

## Abstract

Many Asian countries, including Japan, China, and South Korea, continue to use terms that reference sugar and urine, contributing to ongoing stigma, while most of the rest of the world seem to use terms related to the original “Diabetes,” meaning “to pass through.” The 16th Scientific Meeting of the Asian Association for the Study of Diabetes (AASD) was held, featuring a pivotal joint symposium organized by AASD and the Japanese Association of Diabetes Education and Care where an in‐depth discussion was carried out on diabetes‐related terminology across various Asian countries and regions, with a particular focus on the stigma associated with existing terms. The symposium participants reached a consensus on the necessity of revising the stigmatizing diabetes terminology across Asia and agreed to continue discussions and monitor progress at the 17th AASD Scientific Meeting, scheduled to be held in 2025.

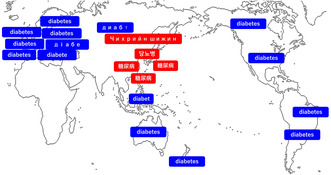

The 16th Scientific Meeting of the Asian Association for the Study of Diabetes (AASD) was held alongside the 7th Ulaanbaatar International Congress of Diabetes and Endocrinology (President: Professor Altaisaikhan Khasag of Mongolian National University of Medical Sciences), featuring a pivotal joint symposium organized by AASD (Chair: Professor Yutaka Seino of Kansai Electric Power Hospital) and the Japanese Association of Diabetes Education and Care (JADEC) (Chair: Professor Yutaka Seino). Chaired by Professors Takashi Kadowaki of Toranomon Hospital and Daisuke Yabe of Kyoto University, the symposium centered on the theme, “What do you call diabetes in your country?” This theme prompted an in‐depth discussion on diabetes‐related terminology across various Asian countries and regions, with a particular focus on the stigma associated with existing terms.

Professor Kadowaki, serving as the Chair of the International Diabetes Federation Western Pacific Region (IDF‐WPR), delivered a keynote address emphasizing the urgent need to address and eliminate the stigma surrounding diabetes through the revision of current terminology. He argued that terms used in Japan, and several Asian countries have significant adverse influences to cause misconceptions and negative perceptions about the disease. Professor Kadowaki introduced a collaborative advocacy initiative led by JADEC and the Japan Diabetes Society (JDS), launched in 2019, which envisions a society where individuals with diabetes feel no need to conceal their condition^1^. This initiative is committed to fostering a correct understanding of diabetes and actively combating the misconceptions and prejudices that fuel stigma^1^. During his presentation, Professor Kadowaki discussed the current Japanese term for diabetes, “糖尿病” (tō‐nyō‐byō), which translates literally to “sugar urine disease.” He presented findings from a JADEC survey involving approximately 1,000 individuals living with diabetes, revealing that 90% of respondents felt discomfort or resistance toward this term, and 80% expressed a strong desire for change. The inclusion of the word “urine” was particularly distressing for many, as it evoked feelings of filthiness and shame. Additionally, the term perpetuates harmful stereotypes, suggesting that diabetes results solely from laziness or poor lifestyle choices. Professor Kadowaki provided a historical overview of the term “diabetes,” tracing its origins to around 150 BC when Aretaeus of Cappadocia coined it from ancient Greek words meaning “to pass through.” Similarly, in 250 BC China, the term “消渴” (xiāo‐kě), meaning “the disappearance or passing away of water or food” was used. Neither of these early terms referenced urine. It was not until the 18th century that “Diabetes Mellitus” meaning “like honey,” emerged in the United Kingdom, and by the 19th century, some European countries transiently co‐used Diabetes Mellitus/Diabetes and terms referencing urine, such as “Pisvloed” (Pis = urine, vloed = flood) in Dutch and “Harnruhr” (Harn = urine, ruhr = dysentery) in German. Japan imported only these urine‐related terms but did not adopt “Diabetes Mellitus/Diabetes” leading to the widespread use of “糖尿病” (tō‐nyō‐byō). While in European countries, these urine‐related terms became obsolete and eventually only the term “Diabetes” was used, many Asian countries continue to use terms that reference sugar and urine, contributing to ongoing stigma (Figure 1). To address this issue, a new Japanese term, “ダイアベティス” (diabetes), written in Katakana (a kind of Japanese character used mainly for loanword = foreign origin word, foreign names and foreign places), has been proposed. This term meets three critical criteria: alignment with academic perspectives, adherence to international standards, and potential to eliminate existing stigma. Professor Kadowaki asserted that adopting “ダイアベティス” is a crucial step toward reducing stigma and aligning Japan with global nomenclature practices. He acknowledged that while a name change alone cannot eradicate all stigma, it significantly contributes to broader efforts aimed at improving societal perceptions of diabetes.

**Figure 1:**
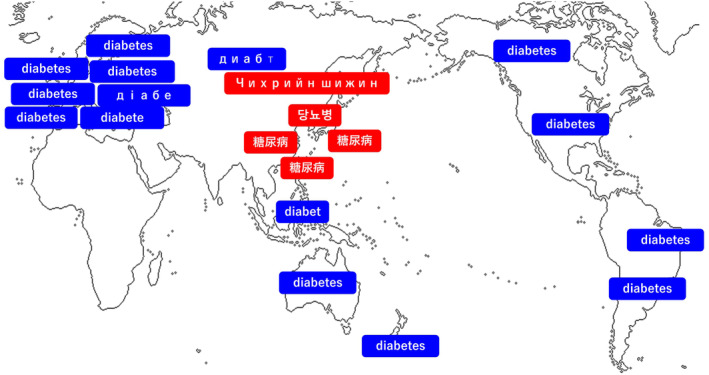
Terminology for “diabetes” across various countries/regions. In many Asian countries/regions, red‐highlighted terms that reference both sugar and urine are prevalent, perpetuating longstanding stigma. For example, Japan uses “糖尿病” (tō‐nyō‐byō), China uses “糖尿病” (tang‐niào‐bìng), South Korea uses “당뇨병” (tang‐nyo‐ppyŏng), and Mongolia uses “ЧИХРИЙН ШИЖИН” (chikhriin shijin). In contrast, most of the rest of the world uses blue‐highlighted terms that are derived from the original “Diabetes,” meaning “to pass through.” For instance, the United Kingdom and the United States use “Diabetes” (dàiəbíːtiːz), Germany uses “Diabetes” (diabétes), France uses “Diabète” (djabεt), and Russia uses “Диабет” (diabet).

Dr. Kazuhiro Tsumura of Kawasaki Municipal Hospital traced the history and evolution of diabetes advocacy efforts in Japan. He highlighted key milestones, including a 100,000‐signature campaign in 1971 advocating for health insurance coverage of insulin injections. These efforts laid the groundwork for improved care and support for individuals with diabetes in Japan. Dr. Tsumura discussed the joint advocacy initiative by JADEC and JDS launched in 2019, focusing on combating misconceptions and prejudices contributing to diabetes stigma^1^. He emphasized that advocacy strategies vary depending on the maturity of healthcare systems: Developing regions prioritize access to standard care, while established systems focus on welfare and stigma elimination. The joint advocacy committee has been instrumental in raising awareness about diabetes‐related stigma in Japan, gathering new scientific data and initiating activities aimed at changing societal perceptions. A significant initiative is the proposed renaming of “糖尿病” to “ダイアベティス” reflecting a more accurate and less stigmatizing understanding of the disease. Dr. Tsumura noted that while “糖尿病” was appropriate when urinary glucose testing was the primary diagnostic method, modern medical understanding necessitates updated terminology. The proposed name has garnered widespread public engagement, evidenced by numerous discussions and comments on social media platforms. Dr. Tsumura concluded that renaming diabetes is a vital step in advancing advocacy efforts and fostering collaboration across Asia.

Professor Chien‐Ning Huang of Chung Shan Medical University discussed efforts to combat diabetes stigma in Taiwan. He introduced the “End Diabetes Stigma and Discrimination” campaign by the Diabetes Association of the Republic of China, which seeks to address challenges individuals face in employment and education due to stigma. He highlighted the potential benefits of changing Taiwan's term for diabetes, also “糖尿病” (tang‐niào‐bìng), drawing parallels to the successful renaming of schizophrenia in Taiwan. Previously known as “精神分裂症” (Jīng shén fēn liè zhèng) implying “split mind”, schizophrenia was renamed “思觉失调症” (sī jué shī tiáo zhèng) to suggest a dysregulation of cognition and perception, effectively reducing stigma. A recent survey indicated that 15.2% of respondents felt “unpleasant” about the term “糖尿病,” suggesting still room for improvement through terminology change.

Professor So Hun Kim of Inha University College of Medicine provided an overview of diabetes awareness and stigma in Korea. Survey findings showed that 86.7% of Koreans consider diabetes a serious health condition, with significant concern about personal risk, especially among older adults and those with higher body mass index. Despite high awareness, individuals with type 1 diabetes often manage their condition in secrecy due to misconceptions that it is congenital or genetic. Disclosure can lead to discrimination in employment and marriage, highlighting deep‐rooted societal prejudices. The current Korean term for diabetes, “당뇨병” (tang‐nyo‐ppyŏng) directly references sugar urine disease, contributing to negative perceptions and misinformation. There is an active movement in Korea to rename diabetes to terms that avoid misunderstanding and prejudice. Advocates propose “췌도부전” (che‐wdo‐bu‐jeon), meaning pancreatic islet insufficiency, as a more accurate and less stigmatizing term. The goal is to adopt terminology that accurately reflects the medical condition without invoking negative connotations.

Professor Weiping Jia and Dr. Yeping Huang of the Shanghai Diabetes Institute discussed the historical and current terminology of diabetes in China. Early terms such as “消渴” and “三消” (sān‐xiāo) described symptoms such as excessive thirst and urination. The modern term “糖尿病” emerged during the Ming and Qing Dynasties, reflecting the discovery of sugar in the urine. They pointed out that research on diabetes‐related stigma in China is limited, but existing studies show that over half of individuals with type 2 diabetes experience stigma manifested through negative stereotypes, devaluation, and discrimination^2^. This stigma is driven by societal beliefs blaming individuals for their condition, perpetuated by harmful language and media portrayals. To mitigate stigma, they advocated for changing the narrative around diabetes, including adopting new terminology and avoiding derogatory terms such as “富贵病” (disease of affluence) and “糖人” (sugar people). They emphasized the need for additional research to understand stigma origins and effective educational and public outreach initiatives to enhance diabetes awareness.

Professor Khasag reported that the Mongolian Diabetes Association recommends adopting the term “Диабет” (diabet), which is “diabetes” in Russian instead of “ЧИХРИЙН ШИЖИН” (chikhriin shijin), which means sugar‐induced polyurea. This shift aligns Mongolia with global practices and aims to reduce associated stigma. Additional comments from attendees, including Dr. Yotsapon Thewjitcharoen from Thailand and Dr. Nagaaki Tanaka from Japan, reinforced the widespread relevance of this issue across Asia. They emphasized that if even a few people experience discomfort with current terminology, it necessitates change. Professor Yabe likened the situation to addressing bullying in schools, stating that action is required even if only a few are affected. Professors Yutaka Seino and Wayne H.H. Sheu, both former Chairs of IDF‐WPR, underscored the importance of changing diabetes terminology in Asian countries to expedite efforts in dismantling‐associated stigmas.

Professor Kadowaki concluded the symposium by affirming that terms for diabetes in many Asian countries, which reference sugar and urine, contribute significantly to stigma and should be urgently revised. Adopting internationally accepted terms expressed in phonetic characters, such as “ダイアベティス, can play a crucial role in reducing negative and stigmatizing perceptions associated with sugar‐ or urine‐related terms, which aligns with global standards. The symposium participants reached a consensus on the necessity of revising the stigmatizing diabetes terminology across Asia and agreed to continue discussions and monitor progress at the 17th AASD Scientific Meeting, scheduled to be held in Taipei in March 2025. This collective effort marks a significant step toward reducing stigma, improving public understanding, and enhancing the quality of life for individuals living with diabetes across the region.

## FUNDING

The authors received no financial support relevant to this article.

## DISCLOSURE

YS has received research funding/grants from Nippon Boehringer Ingelheim Co., Ltd., ARKRAY Marketing, Inc., Taisho Pharmaceutical Co., Ltd., Novo Nordisk Pharma Ltd., Terumo Corporation and Sumitomo Pharma Co.; and consulting/lecture fees from Taisho Pharmaceutical Co., Ltd., Nippon Becton Dickinson Company, Ltd., Novo Nordisk Pharma Ltd., Eli Lilly Japan K.K., Sumitomo Pharma Co., Ltd. and Ono Pharmaceutical Co., Ltd. DY has received consulting/lecture fees from Eli Lilly Japan K.K., Kyowa Kirin Co., Ltd., Nippon Boehringer Ingelheim Co., Ltd., Novo Nordisk Pharma Ltd, Sanofi K.K., and Sumitomo Pharma Co., Ltd.; and research funding/grants from Arkray Inc., the Japan Association for Diabetes Education and Care, Nippon Boehringer Ingelheim Co., Ltd., Novo Nordisk Pharma Ltd., Taisho Pharmaceutical Co., Ltd., and Terumo Corporation. KT has received research funding/grants from the Japan Association for Diabetes Education and Care; and consulting/lecture fees from the Japan Association for Diabetes Education and Care. HCN has received research funding/grants from AstraZeneca, Eli Lilly and Company, Novo Nordisk Pharma Ltd., Sanofi; and consulting/lecture fees from Abbott Laboratories, AstraZeneca, Bayer, Boehringer Ingelheim Co., Ltd., Eli Lilly and Company, Medtronic, Novartis, Novo Nordisk Pharma Ltd., Sanofi. TK has received research funding/grants from Daiichi Sankyo Co., Ltd., Sumitomo Pharma Co., Ltd., Nippon Boehringer Ingelheim Co., Ltd.; and consulting/lecture fees from Taisho Pharmaceutical Co., Ltd., Sumitomo Pharma Co., Ltd., Takeda Pharmaceutical Co., Ltd., Mitsubishi Tanabe Pharmaceutical Co., Ltd., Eli Lilly Japan K.K., Nippon Boehringer Ingelheim Co., Ltd., Novo Nordisk Pharma Ltd., Abott Japan LLC., Teijin Pharma Ltd. SHK, WJ and AK had nothing to declare. YS, DY, WJ and TK are Editorial Board members of the Journal of Diabetes Investigation and co‐authors of this article. To minimize bias, they were excluded from all editorial decision‐making related to the acceptance of this article for publication.

## AUTHOR CONTRIBUTIONS

YS, DY, and TK contributed to the writing of the manuscript and approved the version to be published. KT, C‐NH, SHK, WJ, and AK critically reviewed and revised the manuscript for intellectual content. YS and TK are the guarantors of this work.
